# Potential Applications of Circulating Tumor DNA Technology as a Cancer Diagnostic Tool

**DOI:** 10.7759/cureus.4907

**Published:** 2019-06-16

**Authors:** Anil P Kunnath, Thirujothi Priyashini

**Affiliations:** 1 Department of Applied Biomedical Science and Biotechnology, International Medical University, Kuala Lumpur, MYS

**Keywords:** ctdna, tumor, cancer, diagnostic tool

## Abstract

Cancer is one of the greatest threats posed to society, necessitating appropriate diagnosis methods. Modern targeted therapies have greatly advanced the treatment of several solid tumors. The rational use of these agents requires optimal strategies for the rapid and accurate detection of targetable genomic alterations at the time of initial diagnosis and when acquired resistance to targeted therapies develops. Currently used techniques, such as tissue genotyping, have limitations such as difficulty in categorizing tumors, needing frequent sampling, and difficulty in obtaining samples. To overcome these issues, cost-effective and non-invasive methods are an urgent requisite, which would provide an insight into the real-time dynamics of cancers via circulating biomarkers. Circulating tumor DNA (ctDNA), commonly termed “liquid biopsy,” has emerged as a new, promising non-invasive tool to detect biomarkers in several cancers. The present review aimed to understand the biological concept of ctDNA and its potential as a biomarker in cancer studies and the clinical utility of this evolutionary diagnostic technique.

## Introduction and background

Cancer is one of the major causes of increased mortality due to poor diagnosis at the initial stage of manifestation and difficulties in monitoring the patients along with the therapeutic effect. Previous studies have aimed to identify the novel biomarkers that could resolve the issues, which, in turn, led to the discovery of circulating tumor DNAs (ctDNAs). Ongoing studies on these ctDNAs aim to maximize their utilization as a non-invasive method in cancer patients [1]. Tumor cells undergo alterations or mutations, which are known as somatic alterations, in the genes that are involved in maintaining the normal function of the cells. In the event that these cells divide uncontrollably, fragments of the DNA are released via apoptosis or tumor necrosis into the tissue fluid and the bloodstream. These ctDNAs serve as critical biomarkers due to their specificity towards the mutation forms in the genes of a patient’s cell (not inherited). Thus, the tumor progression can be monitored according to the amount of detectable ctDNAs in a liquid sample, which increases with an increase in tumor stages. Sampling techniques for ctDNAs were reliable in liquid biopsy, which is not only a blood-based detection, but also other easily obtainable fluids, such as urine, spinal fluid, and saliva, can be utilized. The concept of liquid biopsy in the detection of ctDNAs provides an insight in cancer detection, as it was simple, fast, and cost-efficient method for monitoring the cancer status as well as the response of patients to the individualized treatment approach [1].

## Review

Origin, mechanism of ctDNA entry into the bloodstream, and clearance of ctDNA

In a patient with cancer, four origins of circulating DNA might be possible: primary tumor cells, metastatic cells, circulating tumor cells (CTCs), and living “healthy cells” [2]. The mechanism of entry of ctDNA into the bloodstream occurs passively via apoptosis and necrosis of the tumor cells. DNA fragments are obtained from circulating tumor cells as well as via active release, phagocytotic release, and other pathophysiological stress that could be influenced by environmental factors at microscale and post-treatment. The pathway chosen by the cells to release the ctDNA results in various morphologies of the DNA in the blood system. Briefly, apoptosis, which is known as the controlled cellular death mechanism, results in apoptotic bodies that release the ctDNA in the form of the nucleosome, which is proven by typical patterns of fragments (160-200 bp) via electrophoresis [2-3]. Although programmed cell death results in an appropriated own regulation of the nucleosome by phagocytic cells, the elimination process in cancer patients overloaded with the progression of the tumor is correlated with an elevated level of ctDNA along with the circulation of nucleosomes in the blood [2-3]. Necrotic cells release partially digested circulating DNA, and the contribution via this pathway increases as the patient enters the advanced stage of cancer [2-3]. CTCs that escape into the bloodstream evading the phagocytic process contain identical mutational copies of genes as the ctDNA but do not possess the property to metastasize and colonize different sites. These cells undergo lysis and contribute to the circulating DNA content in the blood [2-3]. The active release of the DNA via secretory vesicles of the living cells of cancer, known as exosomes, contributes only slightly towards ctDNA into the blood, suggesting that the pathway via apoptosis and necrosis releases a large portion of ctDNA [3]. Healthy living cells randomly shed DNA as novel replication takes place and is released from the cells into the blood after degradation of a small amount [2-3].

Properties of ctDNA

Length or Size

ctDNA could be single or double-stranded with the variable length depending on the mechanism of cellular release into the blood [2-3]. There are two views regarding the fragment lengths; some reported cases of ctDNA have longer fragments than healthy functioning cells while others reported an opposite concept [2]. Some studies speculated that the apoptotic pathway generates shorter fragments than the other mechanisms while some reviews state that the tumor necrosis pathway results in large fragments [3]. Moreover, the uses of various methods in analytical techniques have resulted in contradictory statements published previously, wherein no clear conclusion could be drawn regarding the length or the size of ctDNA [2-3]. In addition, the fragmentation of DNA is more in a cancer patient as compared to a healthy individual [3].

Stability

In the case of cell-free circulating DNA, the half-life of the fragment is within 16 minutes, but in ctDNA, the half-life of the strand is not yet clarified. The study was carried out based on the kinetics of the tumor DNA; it had a dual phase clearance within one hour and rapid and secondary after 13 hours, respectively [2]. The clearance of cell-free DNA in the blood is via the liver, spleen, and kidney, which increases the search for ctDNA in the urine to understand the potential use of sampling patient’s urine for the detection of the tumor. Moreover, enzymes such as DNases in blood could degrade the circulating DNA, aiding its partial clearance from the blood [3].

Detection methods in routine diagnostics for ctDNA

A variety of potential testing platforms for plasma genotyping have been utilized in the past. Each platform has been validated to a different degree and has been found to exhibit unique capabilities and characteristics. The conventional DNA detection methods, such as Sanger sequencing, lack the sensitivity to detect the low levels of the ctDNA present in the peripheral blood. Thus, the recent assays have utilized an allele-specific quantitative polymerase chain reaction (PCR)-based platform to enrich the mutant DNA or used massive parallel sequencing with the next-generation sequencing (NGS) platform [4]. The most current validated ctDNA assays have been compared against tissue genotyping, and the specificities were consistent between 90% and 100% while the sensitivities variable between 30% and 85%, depending on the test methodology [5].

In sampling ctDNA, the plasma is preferred to serum, as it was found that the lysis of blood cells, which would release cell-free DNA that would interfere with the analytical status of the sample. Such a sample would harbor thousands of copies of circulating free DNA (cfDNA)/mL of plasma, which is diluted because of the presence of both cfDNA and ctDNA, whereas only a small amount of ctDNA is required for detection. Hence, developing methods that could enhance specificity and sensitivity to utilize the ctDNA to its maximum potential is essential. The detection methods are classified into two approaches: target detection for specific alterations in the sequence using PCR, digital (dPCR), and next-generation sequencing, whereas in the untargeted method, the detection of ctDNA is not based on specific mutations or alterations but focuses on whole genome sequencing (WGS) or whole exon sequencing (WES) [6]. Nevertheless, the sensitivity and specificity of detecting ctDNA in cancer mutations by quantitative PCR (qPCR) using different fluorescent probes have not yet been achieved. This leads to the usage of dPCR accompanied by various techniques of detection such as microfluid platform, BEAMing, and droplet-based PCR provided high sensitivity in detecting limited point mutations and genes rearrangements in ctDNA. The only limitation to these techniques was low throughput and constrained multiplexing. Intriguingly, the next generation sequencing technologies detect the broad spectrum of mutations in ctDNA, which involves a variable copy number as well as the detection of single nucleotide variants with high sensitivity. In addition to targeting a single gene or a subset of genes, NGS can also identify genome-wide tumor-derived alterations in ctDNA. The usage of WGS and WES is not cost-effective but reliable for searching the novel biomarker and thus has limited usage [7].

Interpretation of plasma genotyping results

The optimal use and interpretation of plasma genotyping require an understanding of cell-free DNA biology, the assay characteristics of the available technologies, and the application of the tests in each clinical scenario [7]. The clinical utility of plasma genotyping for directing patient care has been extensively studied during initial diagnosis and disease progression in advanced lung cancer, whereas molecular testing results directly impact the treatment-related decision making. On the other hand, tumor tissue genotyping is the standard choice for the detection of potentially targetable alterations. In addition, plasma genotyping might play a role, especially when a tissue biopsy is technically infeasible or the tissue is inadequate to undergo routine genotyping [5].

The biology underlying ctDNA sheds insight into the characteristics of a specific assay required to accurately interpret the ctDNA test results. A majority of plasma genotyping assays might have near-perfect specificity, and sensitivity ranges from 70%-80%, resulting in false-negative tests due to inherent analytic test factors [5]. Thus, a tumor biopsy for confirmatory genotyping should be considered when plasma genotyping results are negative.

Plasma genotyping for resistance mutations

The ability to detect resistance mutations in disease progression is another property of non-invasive testing with plasma genotyping that might be clinically valuable. The detection of EGFR T790M mutations by ctDNA analysis is a key example of this clinical application. Several studies have reported lower specificities for the detection of EGFR T790M by plasma genotyping as compared to tissue genotyping [7]. Thress et al. reported sensitivities of 73% and 81% and specificities of 67% and 58% when using the Cobas and BEAMing platforms, respectively, for the detection of EGFR T790M. The study concluded that the “false-positive” results could be attributed to the tumor heterogeneity of resistance mutations, rather than the lack of specificity of the assay [7]. Subsequent studies demonstrated that patients treated with osimertinib based on a positive plasma test for the EGFR T790M mutation have outcomes similar to those of patients who are treated based on the tissue genotyping results [8]. The recent confirmatory AURA3 study included the patients with T790M-positive non-small-cell lung carcinoma (NSCLC), who had progressed after treatment with at least one EGFR kinase inhibitor. These patients were assigned to either osimertinib or platinum-based chemotherapy and demonstrated outcomes similar to T790M-positive patients by ctDNA testing, thereby supporting the feasibility of using plasma genotyping in this clinical setting [9]. Thus, with respect to the detection resistance mutation, plasma genotyping might be useful to confirm the presence of a targetable resistance mutation such as EGFR T790M. Nonetheless, the sensitivity of plasma genotyping assays is not perfect and, thus, the negative ctDNA test should be conducted using a reflex tumor biopsy. The latter may discover alternative mechanisms of resistance such as the transformation of disease or complex alterations, which cannot be easily identified by plasma genotyping.

Role of ctDNA as a biomarker in cancer treatment and monitoring

The evolution of tumor cells and the chances of recurrence (relapse) in a cancer patient who has undergone surgery and was subjected to treatment are common. Another phenomenon found in cancer patients is that the amount of DNA decreased during the treatment and was not detectable after the surgical removal of the solid tumor [10]. Interestingly, in most cases of cancer relapse, the detectable amount of ctDNA increased and provided an early alert to the patient’s treatment response to cancer, which was similarly observed in the incidence of lung and breast cancer. Strikingly, there was a five to 10-month lead time of relapse in breast cancer before clinical evidence conveyed the same via mammogram by monitoring [11]. In another study, a comparative analysis monitored the tumor progression in the treatment of metastatic breast cancer using ctDNA, CA 15-3, and imaging. Although the latter two methods are the gold clinical standards for monitoring breast cancer treatment, the ctDNA level showed a correlation with the dynamic of tumor burden; in about 40-50% women, the estimation of ctDNA responded to the treatment at an early stage. In patients with lung cancer, the efficiency of the treatment was monitored via an analysis of the anti-EGFR mutation in ctDNA along with imaging (positron-emission tomography), and a correlation was established between the levels and changes of ctDNA, thereby suggesting its potential to evaluate the early identification of cancer relapse [12]. Consequently, the assessment of ctDNA serves as a surveillance biomarker in monitoring the treatment response in real-time, and the recurrence of cancer in a patient enables rapid medical intervention due to early detection.

Role of ctDNA as a biomarker in detecting resistance and mechanism illustration of a therapy

The ability of tumor cells to develop resistance to chemotherapy due to tumor heterogeneity is the major cause of treatment failure in cancer patients [13]. Moreover, tissue biopsy or imaging techniques do not reveal the evolution of cancer genomes during treatment. Hitherto, reliable methods for tracking tumor resistance to therapy have been lacking until recently; the ctDNA was deemed to have the potential to minimize this issue. For instance, in NSCLC patients who received tyrosine kinase inhibitor (TKIs) therapy to epidermal growth factor receptor, the mutation of EGFR has been detected with high specificity (<97%). Also, mapping demonstrated that the patients developed point mutation in exon 21 or deletion in exon 19, which resulted in the resistance. Other mutations, such as the T790M mutation of EGFR, have been detected after the third generation of TKIs treatment in about 45%-50% of the patients who acquired resistance from the previous generation of the drug [13]. Similarly, the colorectal cancer (CRC) patient diagnosed developed resistance to regorafenib via mutations in KRAS, BRAF, and PI3KCA, as detected in ctDNA, whereas those treated with endocrine therapies for breast cancer mutation in ESRI and PIK3CA were detected recently [13]. Hence, the utility of ctDNA is relevant for tracking the evolution of cancer cells, which enables treatment guidelines for the care of cancer patients and reveals the mechanism underlying the treatment resistance. Moreover, several kits have been approved by the Food and Drug Administration (FDA), via sampling ctDNA, for the detection of mutation due to resistance in therapy [13].

Future applications

While the utility of ctDNA analysis for the detection of targetable alterations at the time of diagnosis has been well validated, especially in advanced NSCLC, several other applications of plasma genotyping in cancer are currently being investigated. ctDNA monitoring might be clinically applicable throughout the disease, from pre-diagnosis to advanced disease and during the setting of surveillance as well as the initial diagnosis. These evolving areas require further prospective study before constituting the routine clinical practice; however, they seem promising for the use of precision medicine in oncology.

Early Cancer Detection

Early cancer detection using ctDNA testing represents an emerging area of significant clinical interest [14]. The ability to detect low levels of mutant tumor cfDNA up to two years prior to the initial diagnosis of cancer has been demonstrated previously in clinical studies. Because mutant ctDNA exists only at low concentrations in patients with early-stage disease, highly sensitive assays are required for assessment. The correlation between the tumor stage and the level of detectable ctDNA has been established in several studies, wherein ctDNA was detected in 82%-100% of the patients with stage IV diseases and in 47% of patients with stage I disease [5]. Furthermore, the advances in ctDNA detection have made the early detection of cancer feasible, which is one of the main challenges of implementing ctDNA testing as a screening diagnostic tool; hazards are associated with potential overdiagnosis and false positives. Another limitation and challenge of this approach is that it requires the presence of mutations associated with a high positive predictive value for the diagnosis of cancer. Currently, the interpretation of the detection of mutations in common cancer genes was based on low levels of ctDNA since these might exist even in healthy individuals [15]. For example, the mutations in both TP53 and KRAS have been identified in the skin biopsies of healthy individuals without cancer [16]. Plasma genotyping for cancer detection in the asymptomatic population seems promising; however, further clinical validation is essential before being incorporated into clinical practice.

Minimal Residual Disease and Recurrence Monitoring

Tumor ctDNA might serve as a non-invasive and specific biomarker for predicting the risk of relapse and monitoring the minimal residual disease in patients with early-stage cancer who have undergone curative therapy. Additionally, the ability to risk-stratify the patients following curative surgery or chemotherapy might help define the subsets of patients who are likely to benefit from adjuvant therapy while sparing those who might not require further intensive treatment. This concept was demonstrated in a prospective study consisting of 230 patients with stage II colorectal cancer who underwent ctDNA testing either postoperatively (those who did not receive adjuvant chemotherapy) or after the completion of adjuvant chemotherapy. Also, inferior recurrence-free survival was documented in patients in whom ctDNA was detected postoperatively or after the completion of adjuvant chemotherapy [17]. A similar concept was demonstrated in patients with early-stage breast cancer who had completed curative treatment with neoadjuvant chemotherapy, followed by surgical resection [10]. The detection of ctDNA in the postoperative setting was highly correlated with the risk of relapse. Additionally, the study documented a median lead time of 7.9 months prior to the occurrence of clinical relapse in those who underwent serial monitoring [18].

One challenge in the development of ctDNA assays in this setting is identifying specific alterations in each patient. Several studies have demonstrated the feasibility of developing customized ctDNA panels [19]. The postoperative setting provides an opportunity to utilize residual tumor tissue to identify the patient-specific tumor mutations that might be investigated via personalized plasma ctDNA assays. A recent study in 44 patients with early-stage colorectal cancer utilized residual primary tumor tissue to identify tumor mutations and created patient-specific ctDNA panels that were followed serially throughout the disease. ctDNA was detected before clinical or radiographic relapse in 11/15 patients [20].

Response to Therapy and Metastatic Disease Monitoring

The complete molecular heterogeneity of a tumor cannot be adequately assessed by single or multiple biopsies, whereas a “liquid biopsy” captures genetic information shed from all sites of the disease [10]. The short half-life of ctDNA and the relative ease of serial sampling make ctDNA an ideal biomarker for monitoring response to therapy in the metastatic setting. Mok et al. demonstrated that this was an exploratory analysis of plasma ctDNA samples obtained from patients with advanced NSCLC. These patients were randomized to receive six cycles of gemcitabine and platinum-based chemotherapy plus either sequential erlotinib or placebo [21]. EGFR mutation-specific ctDNA allele levels were quantitated at baseline, after Cycle 3, and at the progression of the disease. A correlation was established between the response rates and survival outcomes in a subset of patients. Progression-free survival and overall survival were significantly longer in patients who had no detectable mutant EGFR alleles as compared to those who had detectable EGFR-mutant alleles after three cycles of treatment. This study provides evidence that changes in the levels of EGFR-mutant alleles might be beneficial for continued treatment with EGFR-targeted therapy in patients with advanced NSCLC, although a further prospective study in larger trials is required [21].

Tool for Drug Development

In early-phase drug development, a non-invasive and rapid method of assessing the disease response and mechanisms of resistance could exert a significant clinical impact. Currently, early-phase clinical trials are time- and resource-intensive, often requiring serial tumor biopsies and frequent imaging in order to obtain the maximal clinical and biological information from each treated patient. Owing to the sensitivity of the current ctDNA platforms, changes in the ctDNA levels might be used to assess the response to treatment as early as several weeks after the initiation of therapy, considerably earlier than when radiographic responses can be used for such assessment. Another potential application of plasma genotyping, especially relevant to targeted therapy trials, is the ability of ctDNA assays to non-invasively monitor for and detect resistance mechanisms in real time. Frenel et al. demonstrated the ability of serial ctDNA monitoring to assess the disease response and clonal evolution in response to targeted therapies in an early-phase clinical trial [22]. Another prime example of the usefulness of plasma genotyping for the detection of novel resistance mechanisms in an early-phase clinical trial setting is the discovery of the EGFR C797S mutation, which mediates resistance to the third-generation EGFR kinase inhibitors. In an exploratory analysis, plasma genotyping was conducted on seven EGFR-mutant patients who were enrolled in the phase I AURA study and who acquired resistance to osimertinib therapy; moreover, an EGFR C797S resistance mutation was detected in one patient. Subsequent analysis of serial plasma samples from patients treated with osimertinib, using a ddPCR (droplet digital PCR) assay, detected an acquired C797S resistance mutation in 6/15 patients, unraveling the resistance mechanism [23].

Genotyping of Other Body Fluids

One of the main advantages of plasma genotyping is its non-invasive nature, which facilitates serial testing over time. ctDNA is also released into the other body fluids and, thus, might be assessed and monitored in these other fluid types. Reckamp et al. demonstrated the feasibility of urine genotyping using a mutation enrichment next-generation sequencing (NGS) platform to compare the rates of detection of EGFR mutations in both urine and plasma against tissue genotyping in EGFR-mutant NSCLC patients receiving rociletinib in the TIGER-X trial. Using tumor tissue as a reference standard, the sensitivity of urine genotyping across the samples was 72% for the detection of T790M mutations, 75% for L858R mutations, and 67% for exon 19 deletions. The sensitivity of urine testing was increased when a higher volume (at least 90-100 mL) of urine was analyzed, similar to those reported with plasma genotyping. In the case of plasma genotyping, urine ctDNA analysis was highly specific, with a specificity of 96% for the detection of T790M mutations, 100% for L858R mutations, and 94% for exon 19 deletions [24]. Thus, urine genotyping might be applicable for detecting and analyzing ctDNA. Recent studies have also explored the possibility of saliva genotyping, which would require a saliva sample of only 20-40 μL to non-invasively detect targetable mutations [25].

The sensitivity of ctDNA assays might play a role in disease detection technology and monitoring bodily fluids as standard testing using cytology exhibited poor sensitivity. For example, ctDNA testing of cerebrospinal fluid (CSF) might be a sensitive means for detecting and monitoring metastatic disease in the central nervous system. The feasibility of such an approach was demonstrated using a dPCR-based assay to quantify the BRAF V600E- or V600K-mutant ctDNA in the CSF of patients with metastatic melanoma or systemic histiocytosis. Mutant ctDNA was detected in 6/11 patients, whereas CSF cytology was positive in only 2/11 patients [26]. Similarly, some studies in the NSCLC population have shown the feasibility of detecting the EGFR mutations in the CSF of patients with known brain metastases [27-28]. Given the difficulty in obtaining tissue from central nervous system tumors, liquid biopsy with ctDNA offers a promising alternative for identifying the mechanisms of disease resistance [29].

Challenges

In a clinical setting, ctDNA genotyping is employed as a diagnostic test, and it is crucial to determine the preanalytical variables and their impact on sensitivity, accuracy, and predictive value. Several different types of assays are currently being explored for clinical validity relative to the concordance between ctDNA measurements, the tumor genotype, and clinical outcomes. A negative ctDNA test result cannot be interpreted easily and can represent either a true-negative result or a tumor that does not shed any ctDNA. Thus, a tumor biopsy should be considered in such cases. ctDNA testing holds significant potential as a tool that can be applied throughout the course of the disease, from cancer detection to monitoring the response to therapy, although routine use in these settings necessitates further investigation.

## Conclusions

Plasma genotyping using ctDNA is a rapidly evolving and promising technology with the potential for significant clinical impact in the field of precision oncology. This blood-based new platform differs in terms of sensitivity, specificity, turnaround time, and the rate of mutation detection. ctDNA is a plasma source of tumor DNA that is investigated for the non-invasive detection and monitoring of tumors. To date, multiple platforms for plasma genotyping have been evaluated to various degrees. Each method has different advantages and limitations, complicating the interpretation and routine integration of these tests into clinical practice. Despite the enormous potential in the field of oncology for the use of liquid biopsy of CTCs and ctDNA and the rapid development and use of tools for comprehensive tumor genome analysis, it cannot yet replace gold standard diagnostic techniques in clinical application. Thus, the harmonization of procedures is essential to create clinical standards validating the liquid biopsy as a clinically relevant biomarker in cancer therapy using a multicentre approach. Nevertheless, validated plasma genotyping assays might be used for positive clinical decisions; however, a completely negative test should still be followed up with reflex tissue genotyping. Inconsistencies in the detection of ctDNA mutations can be improved by approaches that can identify rare mutations with high sensitivity in matched ctDNA and tumor tissue samples. Because of its non-invasive nature, ctDNA testing has potentially promising applications throughout the disease, including early disease detection, risk stratification following curative treatment, and disease response monitoring over a period. Extensive data now support that ctDNA has the potential to be considered as a novel technology in diagnostic areas such as early cancer detection and genotyping in other body fluids. Its application can be extended to therapeutic fields such as drug development research also. These applications require an on-going prospective study before being incorporated into routine care.

## References

[1] Han X, Wang J, Sun Y (2017). Circulating tumor DNA as biomarkers for cancer detection. Genom Proteom Bioinform.

[2] Cheng F, Su L, Qian C (2016). Circulating tumor DNA: a promising biomarker in the liquid biopsy of cancer. Oncotarget.

[3] Thierry AR, El Messaoudi S, Gahan PB, Anker P, Stroun M (2016). Origins, structures, and functions of circulating DNA in oncology. Cancer Metastasis Rev.

[4] Vendrell JA, Mau-Them FT, Béganton B, Godreuil S, Coopman P, Solassol J (2017). Circulating cell free tumor DNA detection as a routine tool for lung cancer patient management. Int J Mol Sci.

[5] Komatsubara KM, Sacher AG (2017). Circulating tumor DNA as a liquid biopsy: current clinical applications and future directions. Oncology (Williston Park).

[6] Ma M, Zhu H, Zhang C, Sun X, Gao X, Chen G (2015). "Liquid biopsy"-ctDNAdetection with great potential and challenges. Ann Transl Med.

[7] Thress KS, Brant R, Carr TH (2015). EGFR mutation detection in ctDNA from NSCLC patient plasma: a cross-platform comparison of leading technologies to support the clinical development of AZD9291. Lung Cancer.

[8] Oxnard GR, Thress KS, Alden RS (2016). Association between plasma genotyping and outcomes of treatment with osimertinib (AZD9291) in advanced non-small-cell lung cancer. J Clin Oncol.

[9] Mok TS, Wu YL, Ahn MJ (2017). Osimertinib or platinum-pemetrexed in EGFR T790M-positive lung cancer. N Engl J Med.

[10] Bagan JV, Scully C (2008). Recent advances in oral oncology 2007: epidemiology, aetiopathogenesis, diagnosis and prognostication. Oral Oncol.

[11] Rodriguez BJ, Córdoba GD, Aranda AG (2018). Plasma sequencing of ctDNA in early stage breast cancer as part of the screening process. J Clin Oncol.

[12] Sun X, Xiao Z, Chen G (2018). A PET imaging approach for determining EGFR mutation status for improved lung cancer patient management. Sci Transl Med.

[13] Fisher R, Pusztai L, Swanton C (2013). Cancer heterogeneity: implications for targeted therapeutics. Br J Cancer.

[14] Fiala C, Diamandis EP (2018). Utility of circulating tumor DNA in cancer diagnostics with emphasis on early detection. BMC Med.

[15] Li G, Sun Y (2007). Liquid biopsy: advances, limitations and clinical applications. JSM Biotechnol Bioeng.

[16] Gormally E, Vineis P, Matullo G (2006). TP53 and KRAS2 mutations in plasma DNA of healthy subjects and subsequent cancer occurrence: a prospective study. Cancer Res.

[17] Babayan A, Pantel K (2018). Advances in liquid biopsy approaches for early detection and monitoring of cancer. Genome Med.

[18] Chae YK, Oh MS (2019). Detection of minimal residual disease using ctDNA in lung cancer. Current evidences and future directions. J Thorac Oncol.

[19] Leary RJ, Kinde I, Diehl F (2010). Development of personalized tumor biomarkers using massively parallel sequencing. Sci Transl Med.

[20] Ng SB, Chua C, Ng M (2017). Individualised multiplexed circulating tumour DNA assays for monitoring of tumour presence in patients after colorectal cancer surgery. Sci Rep.

[21] Mok T, Wu YL, Lee JS (2015). Detection and dynamic changes of EGFR mutations from circulating tumor DNA as a predictor of survival outcomes in NSCLC patients treated with first-line intercalated erlotinib and chemotherapy. Clin Cancer Res.

[22] Frenel JS, Carreira S, Goodall J, Roda D, Perez-Lopez R, Tunariu N (2015). Serial next-generation sequencing of circulating cell-free DNA evaluating tumor clone response to molecularly targeted drug administration. Clin Cancer Res.

[23] Thress KS, Paweletz CP, Felip E (2015). Acquired EGFR C797S mutation mediates resistance to AZD9291 in non-small cell lung cancer harboring EGFR T790M. Nat Med.

[24] Reckamp KL, Melnikova VO, Karlovich C (2016). A highly sensitive and quantitative test platform for detection of NSCLC EGFR mutations in urine and plasma. J Thorac Oncol.

[25] Wei F, Lin CC, Joon A (2014). Noninvasive saliva-based EGFR gene mutation detection in patients with lung cancer. Am J Respir Crit Care Med.

[26] Momtaz P, Pentsova E, Abdel-Wahab O (2016). Quantification of tumor-derived cell free DNA (cfDNA) by digital PCR (DigPCR) in cerebrospinal fluid of patients with BRAFV600 mutated malignancies. Oncotarget.

[27] Zhao J, Ye X, Xu Y (2016). EGFR mutation status of paired cerebrospinal fluid and plasma samples in EGFR mutant non-small cell lung cancer with leptomeningeal metastases. Cancer Chemother Pharmacol.

[28] Yang H, Cai L, Zhang Y, Tan H, Deng Q, Zhao M, Xu X (2014). Sensitive detection of EGFR mutations in cerebrospinal fluid from lung adenocarcinoma patients with brain metastases. J Mol Diagn.

[29] Pentsova EI, Shah RH, Tang J (2016). Evaluating cancer of the central nervous system through next-generation sequencing of cerebrospinal fluid. J Clin Oncol.

